# The double-edged relationship between COVID-19 stress and smoking: Implications for smoking cessation

**DOI:** 10.18332/tid/125580

**Published:** 2020-07-27

**Authors:** Jeroen Bommele, Petra Hopman, Bethany Hipple Walters, Cloé Geboers, Esther Croes, Geoffrey T. Fong, Anne C. K. Quah, Marc Willemsen

**Affiliations:** 1 The Netherlands Expertise Centre for Tobacco Control, Trimbos Institute, Utrecht, The Netherlands; 2 Tobacco Research and Treatment Center, Massachusetts General Hospital, Boston, United States; 3 Department of Health Promotion, Maastricht University, Maastricht, The Netherlands; 4 Department of Psychology, University of Waterloo, Waterloo, Canada; 5 School of Public Health and Health Systems, University of Waterloo, Waterloo, Canada; 6 Ontario Institute for Cancer Research, Toronto, Canada

**Keywords:** COVID-19, smoking, stress, smoking behavior, The Netherlands

## Abstract

**INTRODUCTION:**

Although recent research shows that smokers respond differently to the COVID-19 pandemic, it offers little explanation of why some have increased their smoking, while others decreased it. In this study, we examined a possible explanation for these different responses: pandemic-related stress.

**METHODS:**

We conducted an online survey among a representative sample of Dutch current smokers from 11–18 May 2020 (n=957). During that period, COVID-19 was six weeks past the (initial) peak of cases and deaths in the Netherlands. Included in the survey were measures of how the COVID-19 pandemic had changed their smoking, if at all (no change, increased smoking, decreased smoking), and a measure of stress due to COVID-19.

**RESULTS:**

Overall, while 14.1% of smokers reported smoking less due to the COVID-19 pandemic, 18.9% of smokers reported smoking more. A multinomial logistic regression analysis revealed that there was a dose-response effect of stress: smokers who were somewhat stressed were more likely to have either increased (OR=2.37; 95% CI: 1.49–3.78) or reduced (OR=1.80; 95% CI: 1.07–3.05) their smoking. Severely stressed smokers were even more likely to have either increased (OR=3.75; 95% CI: 1.84–7.64) or reduced (OR=3.97; 95% CI: 1.70–9.28) their smoking. Thus, stress was associated with both increased and reduced smoking, independently from perceived difficulty of quitting and level of motivation to quit.

**CONCLUSIONS:**

Stress related to the COVID-19 pandemic appears to affect smokers in different ways, some smokers increase their smoking while others decrease it. While boredom and restrictions in movement might have stimulated smoking, the threat of contracting COVID-19 and becoming severely ill might have motivated others to improve their health by quitting smoking. These data highlight the importance of providing greater resources for cessation services and the importance of creating public campaigns to enhance cessation in this dramatic time.

## INTRODUCTION

Although current smoking is associated with more severe outcomes of COVID-19^1^, little is currently known about how smokers are responding to the COVID-19 pandemic. A US study conducted on 10 April 2020 found that among smokers who both smoked and vaped, 28% decreased while 30% increased their smoking^2^. A French study conducted between 30 March and 1 April 2020 found that 19% of smokers decreased their smoking and 27% increased their smoking^3^. Research shows that 3.3% of Italian smokers quit smoking during lockdown^4^.

In Poland, 45.2% smokers increased their smoking, 40.0% reported no change, and 14.8 % were unsure if their smoking had changed^5^. A Spanish study reported a non-significant decrease in current smoking prevalence after confinement measures went in effect^6^.

Although these studies show that smokers respond differently to the pandemic, they offer little explanation of why some have increased their smoking, while others decreased it. In this study, we examined a possible explanation for this. As past research has shown that stress during the pandemic might be associated with increase smoking^7^, we investigated the possible role of stress in the pandemic’s impact on this group^8^.

## METHODS

We conducted an online survey among a representative sample of Dutch current smokers from 11–18 May 2020. During that period, COVID-19 was six weeks past the (initial) peak of cases and deaths in the Netherlands. On 11 May 2020, the Dutch government relaxed protection measures by allowing primary schools, libraries, and some small businesses, to re-open^9^.

Participants were recruited via an online panel (I&O Research). This panel invited 5275 potential respondents, of which 2233 completed the screener. Of those, 1067 were eligible for our study (i.e. were current smokers or former smokers who quit smoking in the last year). We analyzed the data of the 975 current smokers in SPSS 25.

We conducted a multinomial logistic regression with change in smoking as dependent variable, using ‘no change’ as a reference category. We included perceived stress due to the pandemic, perceived difficulty of quitting due to the pandemic, and motivation to quit due to the pandemic as predictor variables. All predictors had ‘no change’ as reference category. Finally, we added sex, age, education, and nicotine dependence (Heaviness of Smoking Index) as confounding variables. The Heaviness of Smoking Index (HSI) is a composite measure of nicotine dependence that combines cigarettes smoked per day and time to first cigarette of the day. Higher values of the HSI reflect a greater number of cigarettes smoked and/or a shorter time to first cigarette smoked^10^.

Our survey included items from the Coronavirus Module of the International Tobacco Control Policy Evaluation (ITC) Project surveys being conducted in a number of countries^11,12^. The Central Committee on Research Involving Human Subjects in the Netherlands does not require approval from an ethical review committee for non-medical survey research. All respondents signed an informed consent form.

## RESULTS

The sample of 957 current smokers had a mean age of 45.9 years (SD=16.4), and 43.9% were women. Respondents smoked an average of 10.1 cigarettes per day (SD=7.6) and had an average Heaviness of Smoking Index score of 1.5 (SD=1.5). While 11.9% used e-cigarettes regularly, 6.4% used them every day.

Overall, 14.1% of smokers reported smoking less and 18.9% reported smoking more due to COVID-19. Most frequently reported reasons for smoking less were: living a healthier life (32.3%), being alone more (27.0%), having healthier lungs (20.7%), and being healthier to help with recovery from a potential coronavirus infection (19.4%). Reasons for smoking more were boredom (48.6%), feeling more stressed (43.2%), being alone more (36.6%), and visiting fewer places where smoking is banned (23.5%).

Further, 24.7% of current smokers believed quitting smoking had become more difficult, while 6.4% reported it had become easier since COVID-19. Of current smokers, 16.1% reported being more motivated to quit, while 12.1% reported being less motivated. Further, 50.0% of current smokers reported no stress, 40.1% reported being somewhat stressed, and 9.9% reported being severely stressed as a result of COVID-19.

A multinomial logistic regression analysis revealed that stress was associated with both increased and reduced smoking, independently from perceived difficulty of quitting and motivation to quit (Table 1). Smokers who were somewhat stressed were more likely to have either increased (OR=2.37; 95% CI: 1.49–3.78; p<0.001) or reduced (OR=1.80; 95% CI: 1.07–3.05; p=0.027) their smoking. Severely stressed smokers were even more likely to have either increased (OR=3.75; 95% CI: 1.84–7.64; p<0.001) or reduced (OR=3.97; 95% CI: 1.70–9.28; p<0.001) their smoking. Stress thus appears to affect smokers in different ways; some smokers increase their smoking while others decrease it.

**Table 1:** Multinomial logistic regression for increased and reduced smoking. Adjusted for age, sex, education, and nicotine dependence. Data collected 11–18 May 2020 in the Netherlands (N=957)

*Covariates*	*Indicators*	*Increased smoking*				*Reduced smoking*			

		*b*	*OR*	*95% CI*	*P*	*b*	*OR*	*95% CI*	*P*
**Stress due to the pandemic**	No stress (Ref.)								
	Somewhat stressed	0.86	2.37	1.49–3.78	**<0.001**	0.59	1.80	1.07–3.05	**0.027**
	Severely stressed	1.32	3.75	1.84–7.64	**<0.001**	1.38	3.97	1.70–9.28	**0.001**
**Perceived difficulty of quitting due to the pandemic**	Less difficult	-1.05	0.35	0.03–3.69	0.382	2.39	10.94	4.93–24.30	**<0.001**
	No change (Ref.)								
	More difficult	1.30	3.67	2.28–5.89	**<0.001**	0.06	1.06	0.55–2.04	0.851
**Motivation to quit due to the pandemic**	More motivated	0.48	1.62	0.86–3.04	0.140	1.52	4.57	2.53–8.25	**<0.001**
	No change (Ref.)								
	Less motivated	1.62	5.03	2.74–9.24	**<0.001**	1.45	4.26	1.87–9.70	**0.001**

Results of a multinomial logistic regression analysis with ‘change in smoking’ as dependent variable. Predictor variables were perceived stress due to the pandemic, perceived difficulty of quitting due to the pandemic, and motivation to quit due to the pandemic. Both the dependent variable and the predictors had ‘no change’ as reference category. We added sex, age, education, and nicotine dependence (Heaviness of Smoking Index) as confounding variables.

Smokers who reported less difficulty in quitting were more likely to reduce smoking (OR=10.94; 95% CI: 2.93–24.30; p<0.001) while those reporting greater difficulty were more likely to increase smoking (OR=3.67; 95% CI: 2.28–5.89; p<0.001). Similarly, those who reported greater motivation to quit were more likely to reduce smoking (OR=4.57; 95% CI: 2.53–8.25; p<0.001), and those who reported lower motivation were more likely to increase smoking (OR=5.03; 95% CI: 2.74–9.24; p=0.001). There was one somewhat unexpected finding: lower motivation to quit was also associated with a greater likelihood of reducing smoking (OR=4.26; 95% CI: 1.87–9.70; p<0.001).

## DISCUSSION

Consistent with previous research^2,3^, more current smokers reported having increased rather than decreased their smoking due to COVID-19. Our results suggest that pandemic-related stress is associated with increasing and decreasing smoking. While stress generally makes quitting more difficult^13^, stress resulting from COVID-19 has a mixed effect on motivation to quit. For some, boredom and restrictions in movement might have stimulated smoking^5^. For others, concern about contracting COVID-19 and becoming severely ill might have motivated them to improve their health by stopping smoking^14^.

Less difficulty in quitting and greater motivation to quit were significantly related to smoking reductions due to COVID-19. This highlights the importance of providing greater resources for cessation support services^15^, which are expected to see more smokers with high levels of stress. Indeed, the mounting evidence that smoking is related to severity of COVID-19 illness^1^ could provide greater motivation for smokers to quit. Quitting would potentially help tackling not only smoking-related chronic diseases but also the new infectious-disease threat of COVID-19^16^.

While greater motivation to quit was related to reductions in smoking as expected, so was lower motivation to quit. One possible explanation is that smokers who had reduced their smoking believed that they had already done enough to reduce their risk, and thus had lower motivation to take that more difficult step of quitting altogether. Another possible explanation is that some smokers have reduced their smoking due to fewer social activities, and not because they are motivated to reduce or quit their smoking. The coronavirus confinement measures have limited social smoking (i.e. smoking with friends, at parties, or when going out). As a result, some smokers may have reduced their smoking without it being a motivated decision.

### Limitations

A possible limitation of our study is that since our sample did not include recent quitters, we cannot estimate the possible impact of the pandemic on quitting in the population. Our findings apply to smokers who continued smoking during the pandemic; this limitation also applies to other recent cross-sectional surveys on COVID-19 and smoking^2,3^.

## CONCLUSIONS

Smokers appear to be affected by stress related to the COVID-19 pandemic in different ways: some smokers are smoking more, while others are smoking less. While boredom and restrictions in movement might have stimulated smoking, the concern about contracting COVID-19 and becoming severely ill might have motivated others to improve their health by quitting smoking. This highlights the importance of providing greater resources for cessation support services and the importance of creating public campaigns to enhance cessation in this dramatic time.
